# Hypercholesterolemia and the role of lipid metabolism gene CES1 in immune infiltration promote central nervous system relapse in acute myeloid leukemia

**DOI:** 10.3389/fimmu.2025.1575472

**Published:** 2025-07-23

**Authors:** Wanwan Bao, Yansong Tu, Shan Zhang, Xiaoyan Jiang, Huijun Chen, Huaijun Tu, Jian Li

**Affiliations:** ^1^ The Second Affiliated Hospital, Jiangxi Medical College, Nanchang University, Nanchang, Jiangxi, China; ^2^ The Key Laboratory of Hematology of Jiangxi Province, The Second Affiliated Hospital of Nanchang University, Nanchang, Jiangxi, China; ^3^ Faculty of Science, University of Melbourne, Melbourne, VIC, Australia; ^4^ Clinical Trial Research Center, The Second Affiliated Hospital of Nanchang University, Nanchang, Jiangxi, China; ^5^ The Department of Geratology, The Second Affiliated Hospital of Nanchang University, Nanchang, Jiangxi, China

**Keywords:** central nervous system leukemia, hyperlipidemia, CES1, tumor microenvironment, ESTIMATE, CIBERSORT, acute myeloid leukemia

## Abstract

**Background:**

Alterations in multiple lipid metabolism pathways are associated with cancer progression. However, the relationship between lipid metabolism and central nervous system (CNS) relapse in acute myeloid leukemia (AML) remains unclear.

**Methods:**

We conducted a retrospective analysis of 806 AML cases to evaluate the association between serum lipid levels and the risk of CNS relapse. Additionally, RNA-sequencing data from 895 AML patients were obtained from the TARGET database to identify hub lipid metabolism-related genes (LMRGs) associated with CNS relapse. *In vivo* and *in vitro* experiments were performed to validate the bioinformatics findings.

**Results:**

Patients with CNS relapse exhibited significantly elevated levels of total cholesterol (TC), triglycerides (TG), and low-density lipoprotein cholesterol (LDL-C) compared to the non-CNS relapse group. Hypercholesterolemia was identified as a risk factor for CNS relapse. RNA sequencing of AML patients with or without CNS relapse revealed 1,368 differentially expressed genes (DEGs). Functional enrichment analysis of the DEGs indicated a connection between lipid metabolism and CNS relapse. Through integrating these DEGs, LMRGs, and whole-genome correlation network analysis (WGCNA), carboxysterase 1 (CES1) was identified as a hub LMRG. High CES1 expression was a risk factor for CNS relapse and shorter overall survival. Moreover, CES1 influenced the proportion of nine types of tumor-infiltrating immune cells (TICs), particularly M2 macrophages, as supported by functional studies involving CES1 knockdown and overexpression in AML cells and AML xenograft tumor models.

**Conclusion:**

Hypercholesterolemia and CES1 can promote CNS relapse in AML patients, particularly through CES1’s potential role in modulating immune infiltration within the TME.

## 1 Introduction

Acting as a typical leukemia subtype in adults, acute myeloid leukemia (AML) is a hematologic malignancy characterized by the malignant proliferation of bone marrow stem cells (1). Despite recent advancements in treatment, the mortality rate of AML remains relatively high. Leukemia relapse is one of the most important factors contributing to AML-related deaths, with approximately 50% of patients experiencing relapse after completing a standard course of therapy (2). This implies that around half of AML patients require at least two courses of adjuvant therapy. Central nervous system (CNS) relapse is a rare form of AML relapse that can occur in the early stages of treatment. Clinically, CNS relapse is typically managed with cranial and/or neuraxial irradiation, intrathecal therapy with methotrexate and/or cytarabine, glucocorticoids, and systemic chemotherapy (3, 4). However, even after treatment, patients with CNS relapse still exhibit a 20% reduction in 5-year overall survival and generally have a worse prognosis compared to those without CNS relapse (5). Early administration of intrathecal injections can reduce the risk of CNS relapse in leukemia. Nevertheless, unlike in patients with acute lymphoblastic leukemia (ALL), where intrathecal injections are routinely used as prophylaxis against CNS relapse, such routine prophylaxis is generally not administered to AML patients due to the overall lower risk of CNS relapse (6). This underscores the importance of identifying risk factors for CNS relapse in AML to pinpoint high-risk individuals and implement early interventions.

Significant risk factors for CNS relapse in adult AML have been reported to include age ≤ 45 years, a white blood cell (WBC) count of ≥ 50 × 10^9^/L, and the 11q23 chromosomal abnormality (7). Elevated lactate dehydrogenase levels and chromosome 16 inversion at initial diagnosis have also been associated with CNS relapse in AML (8). Jabbour et al. (9), through a retrospective study, found that in addition to elevated lactate dehydrogenase, the Fms-like tyrosine kinase 3/Internal tandem duplication (FLT3/ITD) mutation could independently predict CNS relapse in AML. Moreover, CNS relapse in ALL has been linked to the upregulation of cholesterol biosynthetic pathways (10). However, limited data are available to address the significant gaps in understanding the relationship between lipid metabolism and CNS relapse in AML.

Dyslipidemia includes hypercholesterolemia, hypertriglyceridemia, decreased HDL-C level, and increased LDL-C level (11). A substantial body of evidence has documented the association of dyslipidemia and tumor development. Patients with chronic viral hepatitis and cirrhosis are at a higher risk of hepatocellular carcinoma when low HDL-C levels are detected (12). An increased risk of head and neck squamous cell carcinoma has also been observed in subjects with higher total cholesterol (TC) and triglyceride (TG) levels (13). Dyslipidemia can also promote cancer metastasis. Specifically, TC and TG are associated with the occurrence of distant metastasis in nasopharyngeal carcinoma (14), esophageal carcinoma (15), gastric carcinoma (16), non-small cell carcinoma (17), colorectal carcinoma (18), and breast cancer (19). Lipid metabolism is altered in tumor cells. Abnormal lipid metabolism—such as enhanced fatty acid production and esterification pathways—occurs due to the rapid expansion of tumor cells, which require increased energy and metabolic resources, thereby promoting tumor growth, invasion, and spread (20, 21). Aberrant lipid metabolism can also impair the host’s immune response to tumors. Disturbances in lipid metabolism may weaken the antitumor immunity by inducing dysfunction in immune cells (e.g., macrophages) (22). Inherited disorders of lipid metabolism also contribute to tumor development. The expression of lipid metabolism-related genes (LMRGs) has been shown to predict prognosis in osteosarcoma and is correlated with the immune microenvironment (TME) (23).

This study aimed to clarify the association between dyslipidemia and CNS relapse in AML and to identify related hub LMRGs, especially their role in immune infiltration within the TME, to provide new insights into the link between lipid metabolism and CNS relapse in AML.

## 2 Methods

### 2.1 Retrospective study

#### 2.1.1 Patients

From February 2006 to July 2022, a total of 1,712 patients with AML were enrolled in our study from the Second Affiliated Hospital, Nanchang University. All patients received standard induction chemotherapy. The observation period ended in January 2023 to ensure at least 6 months of follow-up for accurate assessment of CNS relapse status. This study was approved by the ethics committee of our hospital.

Patients diagnosed with AML according to the WHO (2016) classification criteria for hematopoietic and lymphoid tumors were included. The following exclusion criteria were applied: (1) preexisting diseases associated with hyperlipidemia, e.g., diabetes mellitus, metabolic syndrome, hypertension, hypothyroidism, nephrotic syndrome, and coronary artery disease; and (2) absence of lipid profile data in the serum biochemistry report. Finally, this study included 806 patients.

#### 2.1.2 Clinical data

This study retrieved medical records to access patients’ clinical data, including routine demographic data, WBC counts, serum lipid profiles (TG, TC, HDL-C, LDL-C) at the time of initial consultation, and FAB typing. Dyslipidemia was diagnosed based on the following criteria: TC  ≥ 5.2 mmol/L (hypercholesterolemia), TG  ≥ 1.7 mmol/L (hypertriglyceridemia), HDL-C < 1.0 mmol/L (decreased HDL-C levels), or LDL-C  ≥ 3.4 mmol/L (increased LDL-C levels) (11).

### 2.2 Bioinformatics analysis

#### 2.2.1 Source of RNA-sequencing data

Data from AML cases were downloaded from the Therapeutically Applicable Research to Generate Effective Treatments (TARGET) database (https://ocg.cancer.gov/programs/target) (August 2023). After excluding (1) duplicate patients, (2) cases without CNS relapse information, and (3) those without RNA-sequencing (RNA-seq) data, a total of 895 AML cases were included for transcriptomic RNA-seq analysis. TPM values from RNA-seq were log2-transformed.

#### 2.2.2 Identification of DEGs

DEGs were identified using the DESeq2 package (version 3.40.2) in R, based on the criteria of “adjusted *p*-value < 0.05 and | log2 (FC) | > 1”.

#### 2.2.3 Functional enrichment analysis

DEGs were subjected to Gene Ontology (GO), Kyoto Encyclopedia of Genes and Genomes (KEGG) pathway enrichment analyses, and Gene Set Enrichment Analysis (GSEA) using the clusterProfiler and pathview R packages. Significant GO and KEGG pathway enrichment was defined by *p*-values < 0.01 and FDR < 0.05. The GO analysis includes biological process(BP), cellular component(CC), and molecular function(MF). For GSEA, enrichment was considered significant based on the criteria of |NES| > 1, NOM *p*-value < 0.05, and FDR (*q*-value) < 0.25.

#### 2.2.4 Immune microenvironment evaluation

The ESTIMATE algorithm (R 3.5.1) was used to estimate the proportion of immune and matrix components in the TME for each sample. This enabled the determination of the immune contribution to the TME, quantified as the immune score.

For the obtained transcriptome data, we used the deconvolution algorithm-based CIBERSORT to estimate the composition and abundance of immune cells. This study evaluated the effect of CES1 on the proportions of 22 immune cell types in AML.

#### 2.2.5 WGCNA-based screening of key modules

The gene expression matrix, consisting of genes (*p* < 0.05) from differential analysis, was used for whole-genome correlation network analysis (WGCNA) in the R package to investigate correlations with CNS relapse. Outlier samples were identified through hierarchical clustering, after which a scale-free network was constructed. An adjacency matrix (*β* = 16, *R* ^2^ = 0.9) was generated and transformed into a topological overlap matrix (TOM). Gene modules (module eigengenes [ME]) were identified through hierarchical clustering based on 1-TOM and were merged if their ME correlation exceeded 0.75. Based on Pearson correlation analysis with clinical features, genes from key modules most strongly associated with CNS relapse were selected for further analysis. It is important to note that the grey module represents genes that do not belong to any specific module.

### 2.3 *In vivo* and *in vitro* experiments

*In vivo* and *in vitro* experiments are detailed in the **Supplementary Data**.

### 2.4 Statistical analysis

**Figure 1:**
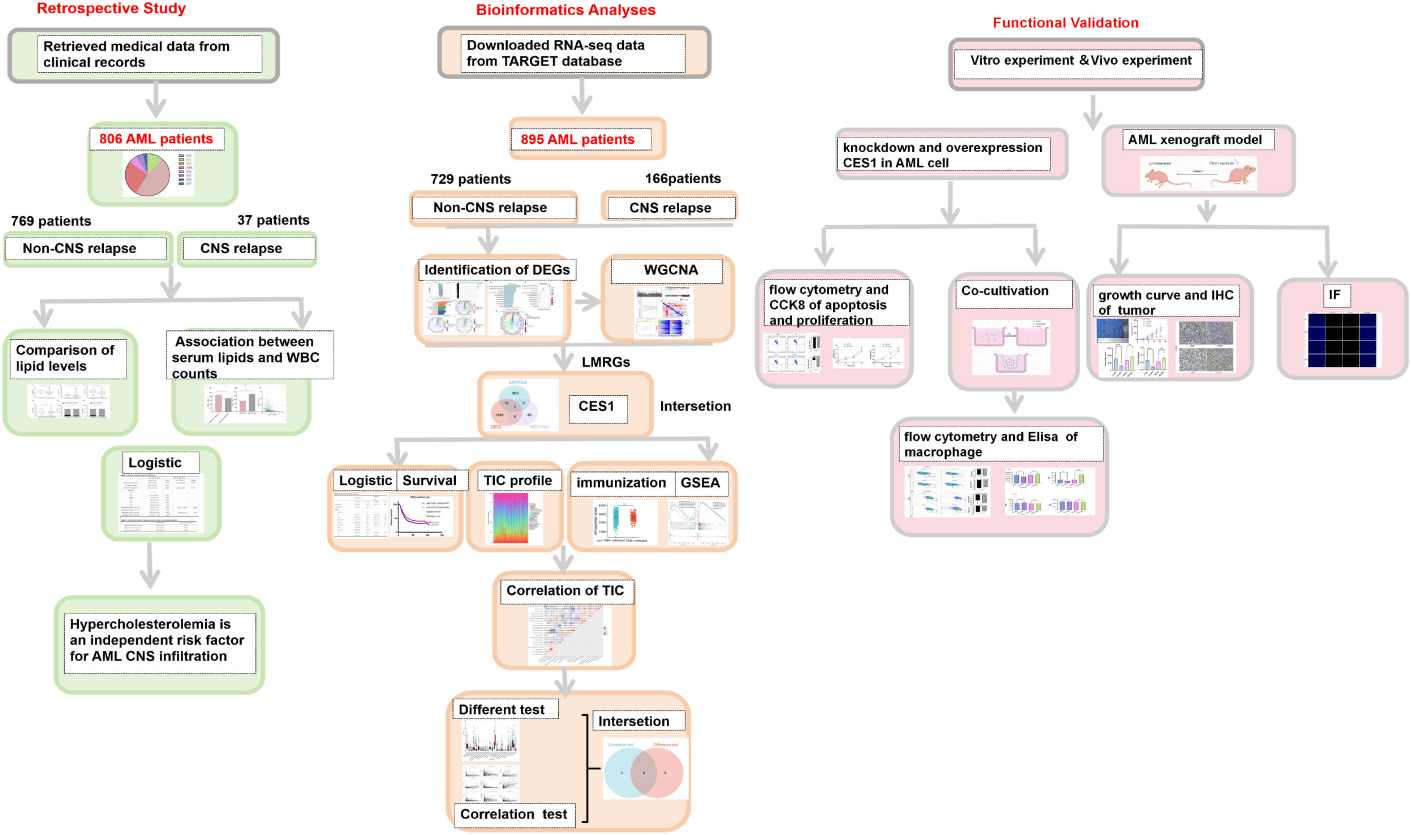
Schematic overview of the analysis workflow used in this study.

Normally distributed variables were compared using the *t*-test (mean ± standard deviation [SD]), while nonnormally distributed variables (interquartile ranges [IQR]) were compared using the Mann–Whitney *U* test. Categorical variables (%) were analyzed using the Chi-square test. Linear regression was employed to assess the relationships between TG, HDL-C, and WBC counts. A binary logistic regression model was used to identify independent risk factors for CNS relapse. A two-tailed *p*-value < 0.05 was considered statistically significant. Kaplan–Meier survival curves were evaluated using the log-rank test. The schematic diagram was created using FigDraw (ID: OITSWfc141). **Figure 1** is intended to visually summarize the analysis workflow of this study

## 3 Results

### 3.1 Comparison of baseline clinical characteristics and laboratory indicators between the CNS relapse and non-CNS relapse group

**Figure 2:**
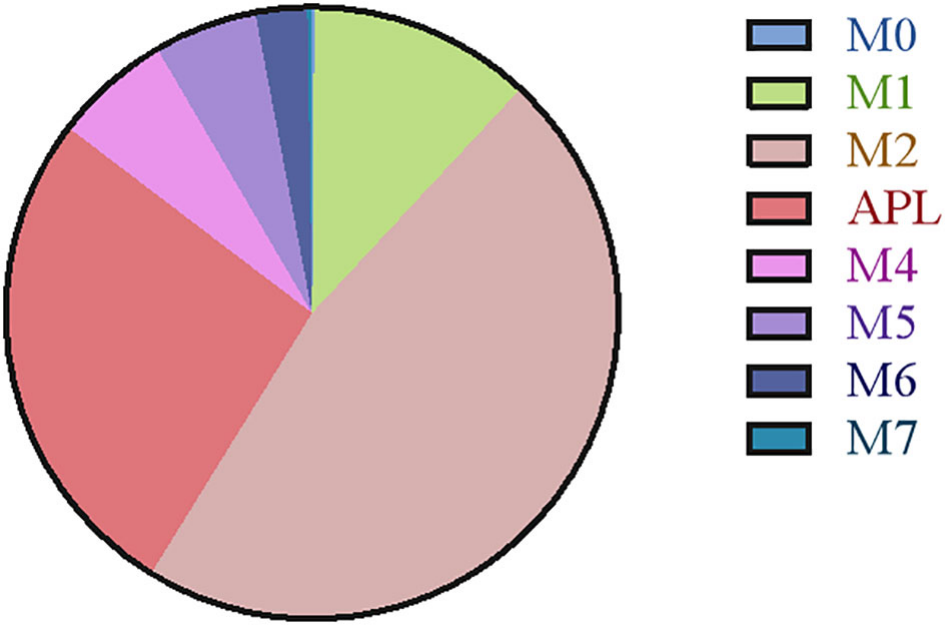
Pie charts illustrating the categorization of different FAB subtypes.

The retrospective study included 806 AML patients, of whom 37 had CNS relapse and 769 had non-CNS relapse. As summarized in **Table 1**, the mean age was 56 years, with 419 (54.5%) men. No differences were detected in sex, hemoglobin, and FAB subtypes (all *p* >  0.05). However, compared to the non-CNS relapse group, the CNS relapse group presented with lower mean age (*p* = 0.001) and platelets (*p* < 0.001). The CNS relapse group also exhibited a significantly higher WBC count (*p* = 0.026), consistent with previous reports (6). **Figure 2** shows the distribution of AML cases by FAB subtype, with M2 being the most common (46.9%).

**Table 1:** Patient characteristics.

Characteristics	Total (*n* = 806)	Non-CNS relapse (*n* = 769)	CNS relapse (*n* = 37)	*p*-value
Age (years, median [IQR])	56.0 (43.0, 68.0)	56.0 (43.0, 68.0)	46.0 (29.0, 59.0)	**0.001**
Sex (*n* (%))				0.059
Male	445 (55.2)	419 (54.5)	26 (70.3)	
Female	361 (44.8)	350 (45.5)	11 (29.7)	
Hemoglobin (g/L, median [IQR])	72 (58.0, 93.0)	72.0 (58.0, 92.0)	73.0 (59.0, 95.0)	0.412
Platelets (g/L, median [IQR])	33 (16, 79)	35.0 (17.0, 81.0)	20.0 (8.0, 27.0)	**< 0.001**
White blood cell (× 10^9^/L, median [IQR])	5.66 (2.38, 28.32)	5.56 (2.27, 27.59)	12.16 (4.30, 59.0)	**0.026**
WBC ≥ × 10^9^/L (*n*; %)	144 (17.866)	132 (17.165)	12 (32.43)	**0.032**
LDL-C (mmol/L, median [IQR])	2.09 (1.62, 2.56)	2.07 (1.61, 2.54)	2.40 (1.92, 3.05)	**0.004**
TG (mmol/L, median [IQR])	1.43 (1.05, 1.97)	1.42 (1.04, 1.96)	1.68 (1.22, 2.34)	**0.029**
TC (mmol/L, median [IQR])	3.61 (2.98, 4.22)	3.60 (2.96, 4.18)	4.20 (3.35, 4.97)	**< 0.001**
HDL-C (mmol/L, median [IQR])	0.80 (0.63, 1.01)	0.80 (0.63, 1.02)	0.77 (0.61, 0.91)	0.249
Hypercholesterolemia (*n*; %)	34 (4.21)	28 (3.64)	6 (16.21)	**< 0.001**
Hypertriglyceridemia (*n*; %)	278 (34.49)	262 (34.07)	16 (43.24)	0.252
Decreased HDL-C levels (*n*; %)	595 (73.82)	563 (73.21)	32 (86.48)	0.073
Increased LDL-C levels (*n*; %)	22 (2.73)	18 (2.34)	4 (10.81)	**0.002**
FAB (*n*; %)				
M0	1 (0.1)	1 (0.1)	0 (0.00)	–
M1	95 (11.8)	91 (11.8)	4 (10.8)	0.851
M2	378 (46.9)	362 (47.1)	16 (43.3)	0.648
APL	214 (26.5)	202 (26.3)	12 (32.4)	0.407
M4	50 (6.20)	48 (6.2)	2 (5.4)	0.837
M5	44 (5.5)	42 (5.5)	2 (5.4)	0.988
M6	22 (2.7)	21 (2.7)	1 (2.7)	0.992
M7	2 (0.3)	2 (0.3)	0 (0.0)	–

Bold values indicate statistical significance.

*WBC*, white blood cells; *IQR*, interquartile range; *TC*, total cholesterol; *TG*, triglyceride; *HDL-C*, high-density lipoprotein cholesterol; *LDL-C*, low-density lipoprotein cholesterol; *M0*, acute myeloid leukemia micronized; *M1*, acute granulocytic leukemia undifferentiated; *M2*, acute granulocytic leukemia partially differentiated; *APL*, acute promyelocytic leukemia; *M4*, acute granule-monocytic leukemia; *M5*, acute monocytic leukemia; *M6*, erythroleukemia; *M7*, acute megakaryocytic leukemia.

**Figure 3:**
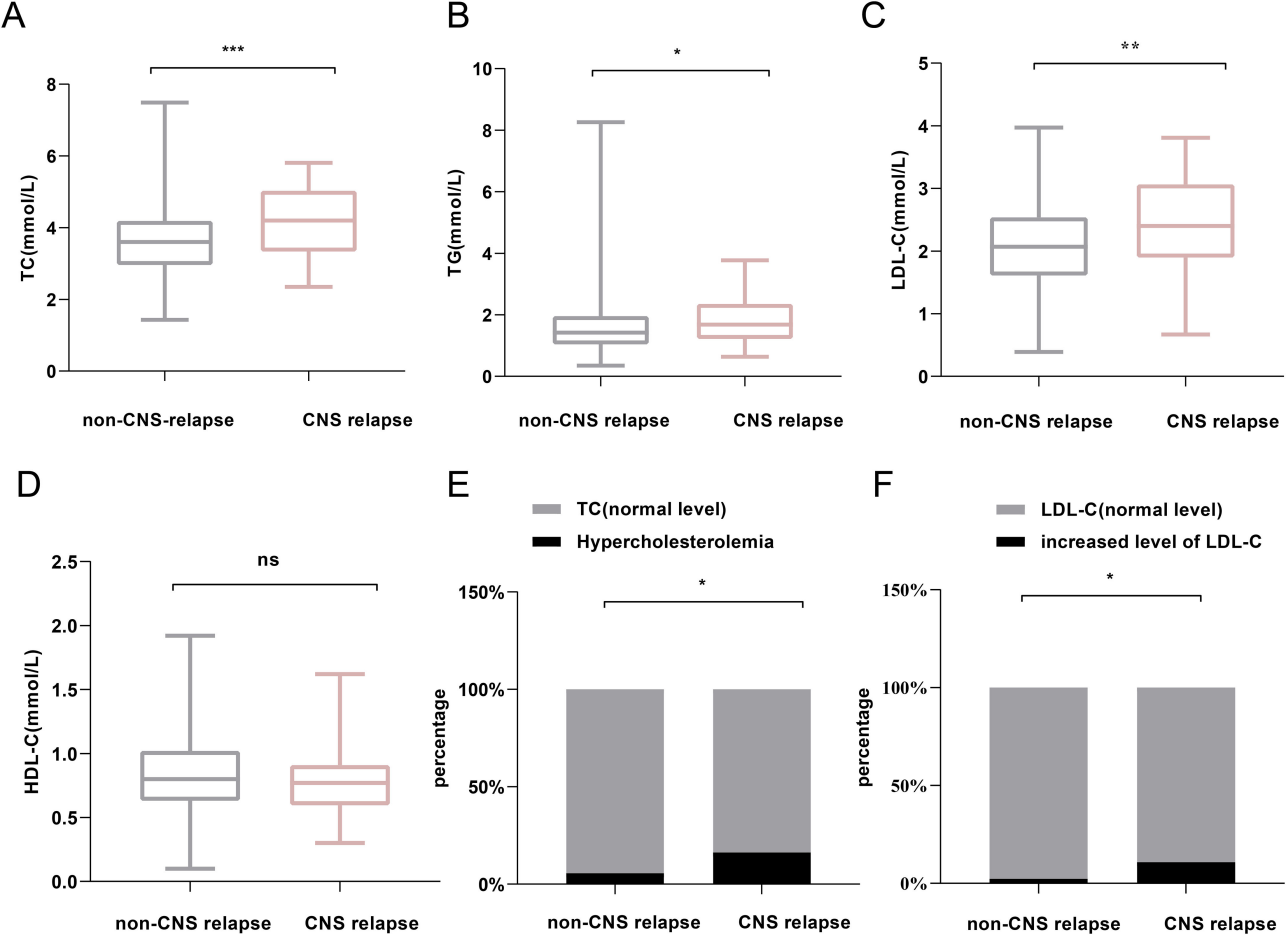
Comparison of lipid profiles between non-CNS relapse and CNS relapse groups. **(A)** TC levels. **(B)** TG levels. **(C)** LDL-C levels. **(D)** HDL-C levels. **(E)** Prevalence of hypercholesterolemia. **(F)** Prevalence of increased LDL-C level. ^*^ *p* < 0.05; ^**^ *p* < 0.01; ^***^ *p* < 0.0001; ns, not significant.

Regarding serum lipid profiles, the CNS relapse group had significantly higher levels of TC (*p* < 0.001), TG (*p* = 0.029), and LDL-C (*p* = 0.004) (**Figures 3A–D**). Both groups had HDL-C levels below the normal range (1.0 mmol/L), although there was no significant difference between them. Moreover, the CNS relapse group had a higher prevalence of hypercholesterolemia (*p* < 0.001) and elevated LDL-C levels (*p* = 0.002) (**Figures 3E, F**).

In addition, we stratified AML patients into non-APL and APL subgroups. Consistent with a previous report (24), lipid levels were generally higher in the APL group compared to the non-APL group (**Supplementary Figure S1**). Given this, we analyzed lipid distributions in the non-CNS relapse and CNS relapse groups within both the non-APL and APL subgroups. Both subgroups showed consistent trends in lipid differences between the non-CNS relapse and CNS relapse groups (**Supplementary Figures S2, S3**), although some differences did not reach statistical significance, particularly in the APL group (likely due to the reduced number of CNS relapse cases after grouping). Furthermore, MANOVA analysis showed no significant interaction effect between APL subtype and CNS relapse status (**Supplementary Table S1**), indicating that the APL subtype is not a confounding factor in the observed lipid differences between the non-CNS relapse and CNS relapse groups.

### 3.2 Association between serum lipids and WBC counts

**Figure 4:**
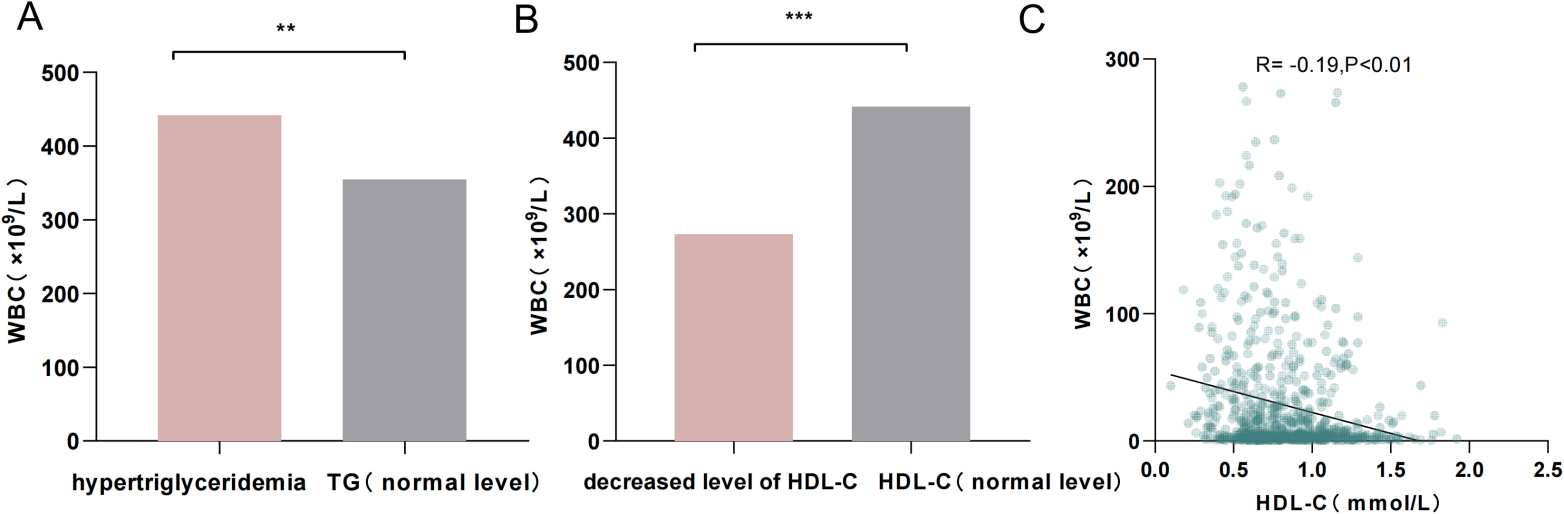
**(A)** Comparison of WBC counts between patients with hypertriglyceridemia and those with normal TG levels. **(B)** Comparison of WBC counts between patients with decreased HDL-C levels and those with normal HDL-C levels. **(C)** Linear scatterplot showing the correlation between HDL-C levels and WBC counts. ^**^ *p* < 0.01; ^***^ *p* < 0.001.

Patients with hypertriglyceridemia had higher WBC counts (7.61 × 10^9^/L vs. 5.18 × 10^9^/L, *p* = 0.01; **Figure 4A**), and those with decreased levels of HDL-C also exhibited higher WBC counts (5.63 × 10^9^/L vs. 4.26 × 10^9^/L, *p* < 0.001; **Figure 4B**). Therefore, linear regression analysis was performed to assess the relationships between TG, HDL-C, and WBC counts. No linear correlation was found between TG and WBC counts. However, HDL-C levels showed a negative correlation with WBC counts (*R* ^2^ = − 0.19, *p* < 0.01; **Figure 4C**).

### 3.3 Serum lipid levels and CNS relapse risk

Regression analysis was further conducted in 803 patients after excluding one case of M0 and two cases of M7 based on FAB typing due to their limited significance. As shown in **Table 2**, univariate logistic analysis identified age ≤ 45 years, WBC count ≥ 50 × 10^9^/L, hypercholesterolemia, and increased LDL-C levels as significant factors for CNS relapse. Multivariate analysis revealed that only WBC count ≥ 50 × 10^9^/L (OR = 2.33, *p* = 0.023), hypercholesterolemia (OR = 4.83, *p* = 0.006), and age ≤ 45 years (OR = 2.35, *p* = 0.014) were independent risk factors for CNS relapse. Considering sex and APL subtype differences in serum lipids, the multivariate analysis results remained consistent for sex and APL subtype (**Table 3**).

**Table 2:** Binary logistic regression analysis results.

Variables	Univariable analysis		Multivariable analysis	
	OR (95% CI)	*p*-value	OR(95% CI)	*p*-value
Age ≤ 45 years (yes/no)	2.18 (1.12, 4.23)	0.021	2.35 (1.18, 4.65)	0.014
Sex (male/female)	1.99 (0.97, 4.08)	0.061		
WBC ≥ 50 × 10^9^/L (yes/no)	2.316 (1.135, 4.727)	0.021	2.33 (1.12, 4.84)	0.023
FAB type				
M1	1			
M2	1.01 (0.33, 3.08)	0.992		
APL	1.35 (0.42, 4.30)	0.610		
M4	0.95 (0.17, 5.36)	0.952		
M5	1.08 (0.19, 6.15)	0.928		
M6	1.08 (0.12, 10.20)	0.944		
Hypercholesterolemia (yes/no)	5.10 (1.97, 13.22)	0.001	4.83 (1.55, 15.02)	0.006
Hypertriglyceridemia (yes/no)	1.47 (0.76, 2.87)	0.254		
High LDL-C levels (yes/no)	5.06 (1.626, 15.78)	0.005	2.08 (0.54, 7.97)	0.283
Low HDL-C levels (yes/no)	2.35 (0.91, 6.12)	0.079		

**Table 3:** Multivariable logistic regression results after adjusting for gender and APL.

Variables	OR (95% CI)	*p*-value (corrected sex and APL)
Hypercholesterolemia (yes/no)	6.954 (2.478, 19.447)	< 0.001
WBC ≥ 50 × 10^9^/L (yes/no)	2.467 (1.151, 5.287)	0.020
Age ≤ 45 years (yes/no)	2.465 (1.215, 5.002)	0.012

### 3.4 Identification of DEGs of CNS relapse in AML

**Figure 5:**
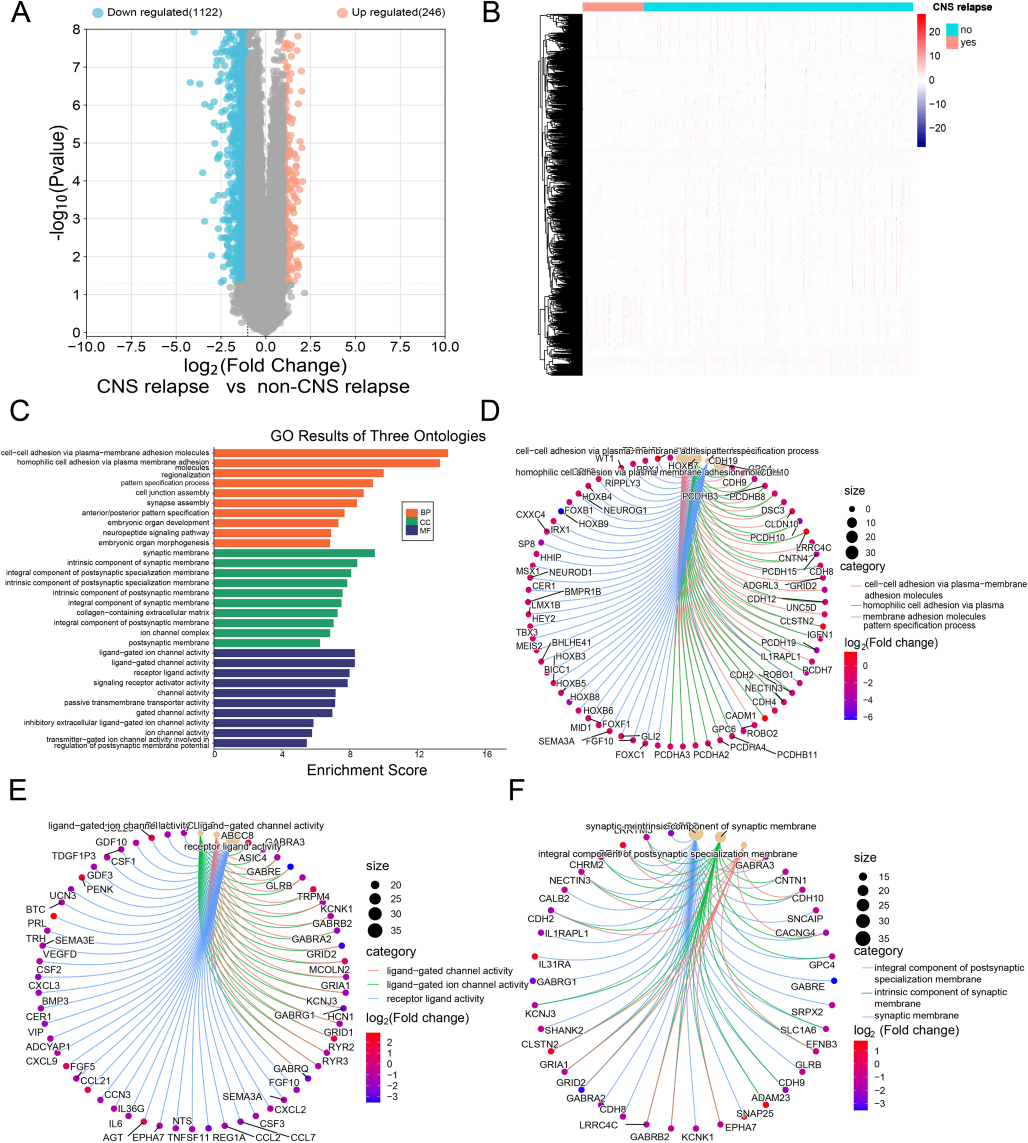
**(A)** Volcano plot showing downregulated genes in blue and upregulated genes in red. **(B)** Heatmap of DEGs. **(C)** GO functional enrichment histograms. **(D)** Chord plot of the top 3 enriched BP terms. **(E)** Chord plot of the top 3 enriched MF terms. **(F)** Chord plot of the top 3 enriched CC terms.

**Figure 6:**
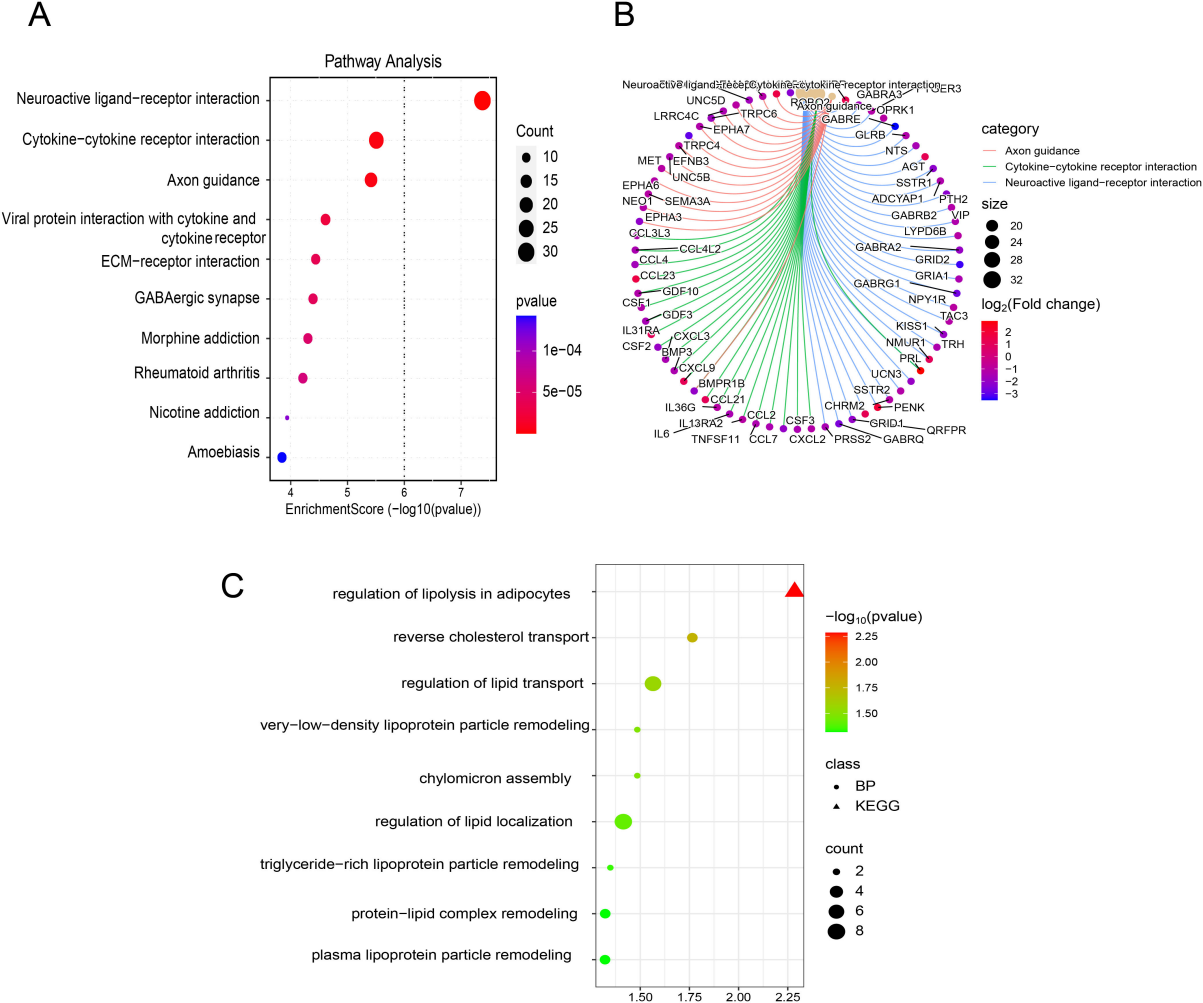
**(A)** Bubble plot of KEGG enrichment analysis. **(B)** Chord plot of KEGG enrichment analysis. **(C)** Bubble plot of enrichment results related to lipid metabolism from BP and KEGG pathway analyses.

Based on RNA-seq profiling from the TARGET database, 1,368 DEGs were identified between the CNS relapse (*n* = 166) and non-CNS relapse (*n* = 729) groups. A heatmap and volcano plot showed that 1,122 genes were downregulated, while 246 genes were upregulated (**Figures 5A, B**). Furthermore, in **Figures 5C–F**, the BP terms were primarily involved in cell–cell adhesion via plasma membrane adhesion molecules, homophilic cell adhesion via plasma membrane adhesion molecules, regionalization, etc. The CC terms were primarily associated with the synaptic membrane, intrinsic components of the synaptic membrane, and integral components of the postsynaptic specialization membrane. The MF terms included receptor–ligand activity, ligand-gated ion channel activity, ligand-gated channel activity, etc. KEGG analysis revealed abnormal alterations in several signaling pathways during CNS relapse, including neuronal ligand–receptor interactions, cytokine–receptor interactions, synaptic guidance, hematopoietic cell pathways, and chemokine signaling pathways (**Figures 6A, B**). Notably, some DEGs were enriched in lipid metabolism-related BP, including reverse cholesterol transport, plasma lipoprotein particle remodeling, triglyceride-rich lipoprotein particle remodeling, and very-low-density lipoprotein particle remodeling. Additionally, KEGG pathway analysis highlighted enrichment in the regulation of lipolysis in adipocytes (**Figure 6C**). These findings support the potential role of lipid metabolism abnormalities in CNS relapse.

### 3.5 WGCNA and identification of key module and hub LMRGs

**Figure 7:**
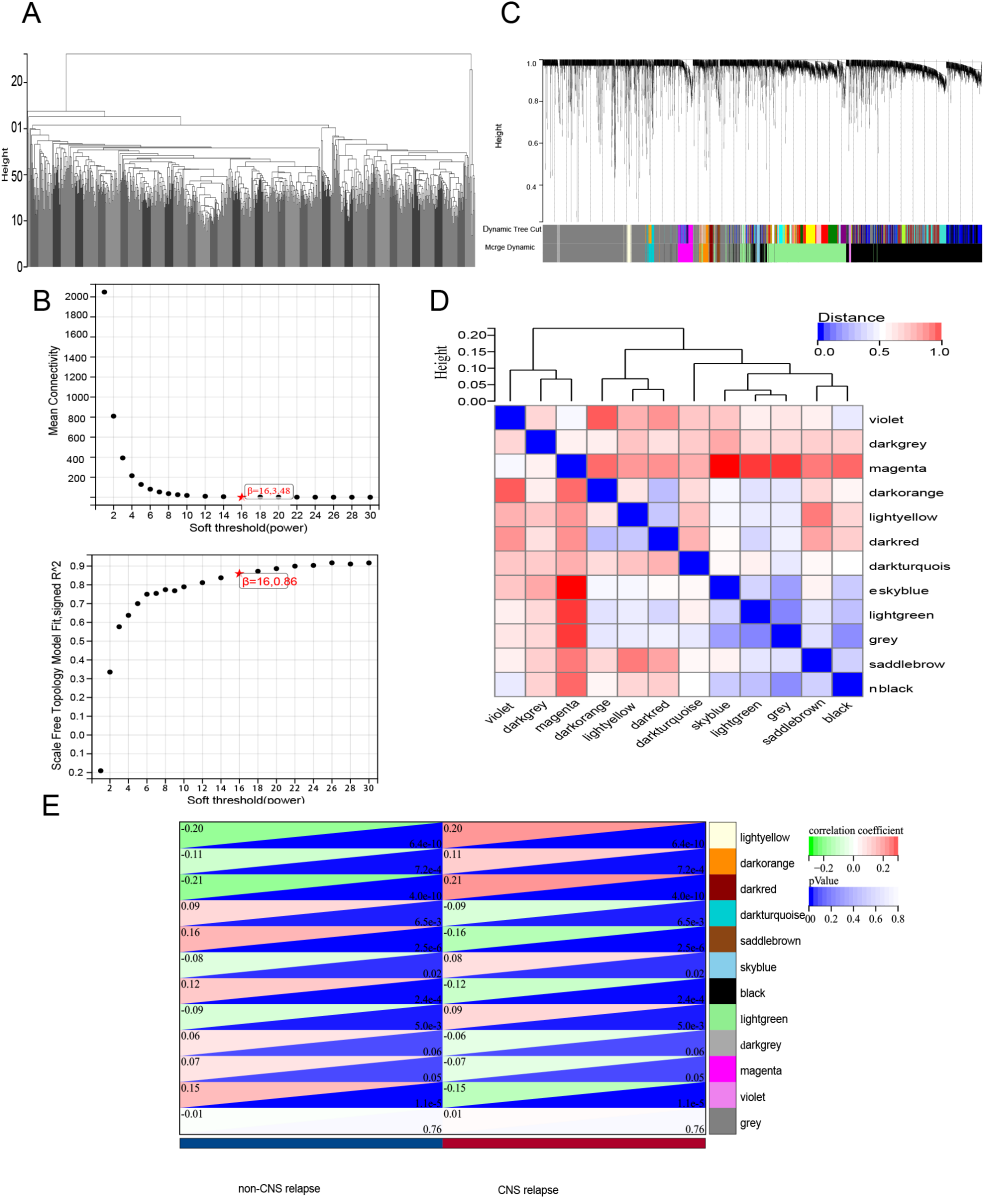
WGCNA. **(A)** Sample clustering analysis. **(B)** Assessment of soft-threshold power. Top, the average connectivity at various soft-threshold powers; bottom, the scale-free fit index corresponding to each power. **(C)** Gene clustering dendrogram based on the dissimilarity measure (1-TOM), showing module assignment with highly coexpressed genes grouped together. **(D)** Clustering of module eigenvector genes. **(E)** Heatmap showing the correlation between gene modules and CNS relapse.

**Figure 8:**
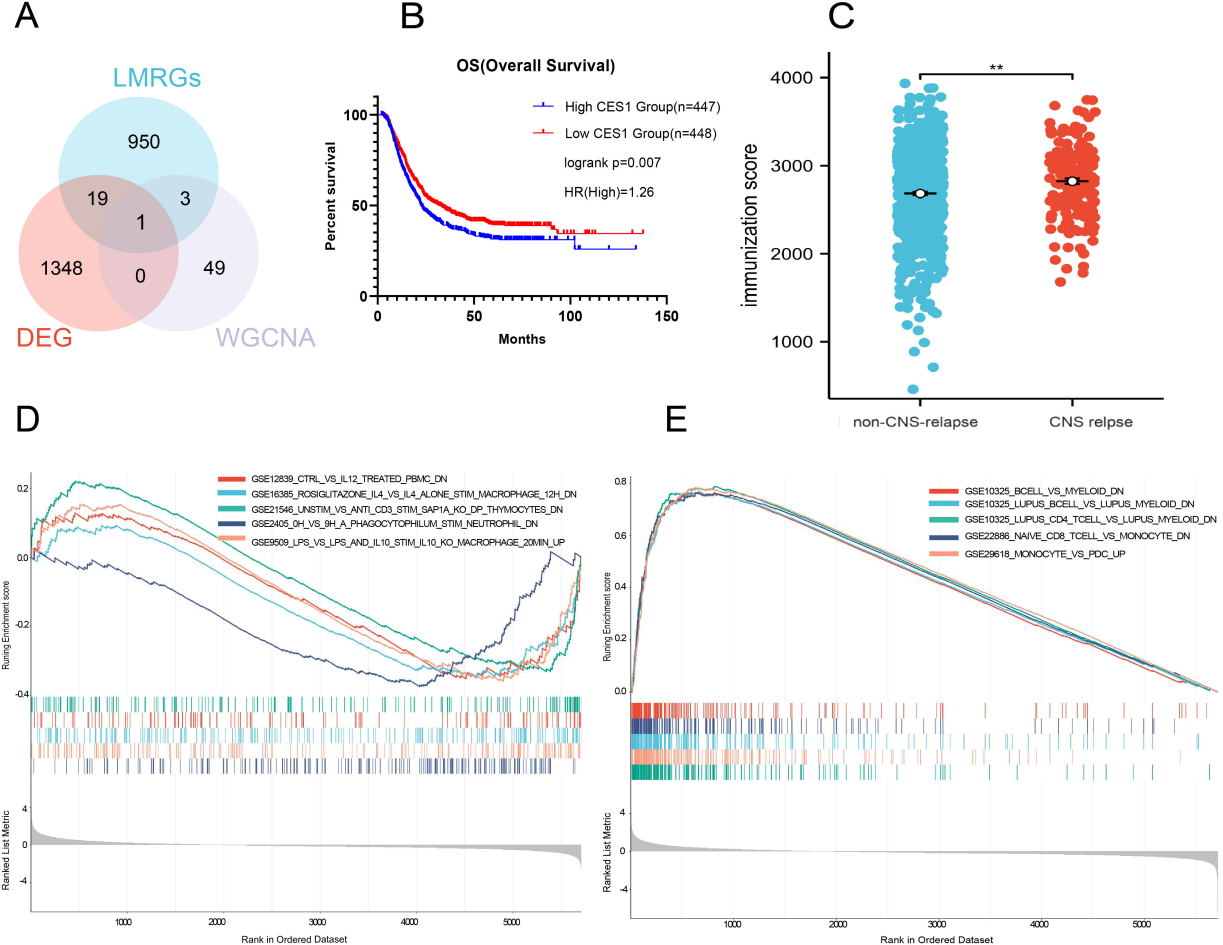
**(A)** Wayne diagram showing the intersection of DEGs, LMRGs, and WGCNA modules. **(B)** Survival analysis comparing high CES1 and low CES1 expression groups. **(C)** Comparison of immune scores between non-CNS relapse with CNS relapse. **(D)** Gene set enrichment analysis of low CES1 expression samples in the C7 ImmuneSigDB collection. **(E)** Gene set enrichment analysis of high CES1 expression samples in the C7 ImmuneSigDB collection.

First, hierarchical cluster analysis was performed on the samples to identify outliers, which were subsequently removed (**Figure 7A**). Next, the soft-threshold power was determined to be 16 (scale-free *R* ^2^ = 0.85) (**Figure 7B**). WGCNA analysis identified a total of 12 modules (**Figures 7C, D**). Among these, the dark red module exhibited the strongest correlation with CNS relapse and was selected for further analysis (**Figure 7E**). This study retrieved 973 LMRGs from GSEA (**Supplementary Table S2**). Further intersection analysis identified CES1 as the hub LMRG (**Figure 8A**).

### 3.6 Prognostic analysis of hub LMRGs

To investigate whether CES1 affected CNS relapse in AML, 865 individuals were included for regression analysis after excluding 30 individuals with missing information. Univariate logistic analysis identified the significance of high-risk group status, WBC count ≥ 50 × 10^9^/L, age ≥ 18 years, FLT3/ITD positive and NPM mutations as significant factors for CNS relapse. Furthermore, multivariate analysis showed that age ≥ 18 years was an independent protective factor (OR = 0.95, *p* = 0.004), while WBC count ≥ 50 × 10^9^/L (OR = 2.02, *p* < 0.001) and high CES1 expression (OR = 2.20, *p* < 0.001) were independent risk factors (**Table 4**). These results suggest that the risk of CNS relapse is 2.2-fold higher in patients with high CES1 expression compared to those with low CES1 expression. In addition, Cox regression analysis revealed that patients with high CES1 expression had shorter overall survival than those with low CES1 expression (**Figure 8B**).

**Table 4:** Binary logistic regression analysis results.

Variables	Univariable analysis		Multivariable analysis	
	OR (95% CI)	*p*-value	OR (95% CI)	*p*-value
CES1 (high/low)	2.52 (1.76, 3.62)	< 0.001	2.20 (1.47, 3.30)	< 0.001
Sex (male/female)	1.31 (0.92, 1.85)	0.128		
Risk group				
Standard risk	1			
Low	0.91 (0.61, 1.37)	0.676	1.10 (0.57, 2.14)	0.761
High	0.55 (0.30, 0.98)	0.044	0.82 (0.39, 1.72)	0.599
WBC ≥ 50 × 10^9^ (yes/no)	1.98 (1.41, 2.80)	< 0.001	2.02 (1.39, 2.93)	< 0.001
FLT3/ITD	0.60 (0.37, 0.99)	0.048	0.84 (0.44, 1.61)	0.609
NPM (yes/no)	0.13 (0.18, 0.96)	0.046	0.17 (0.02, 1.40)	0.101
CEBPA	1.31 (1.58, 2.96)	0.508		
MLL (yes/no)	0.98 (0.66, 1.47)	0.955		
Age ≥ 18	0.94 (0.91, 0.97)	< 0.001	0.95 (0.92, 0.98)	0.004
Inv6	2.21 (1.33, 3.69)	0.002	1.40 (0.63, 3.13)	0.401

### 3.7 Immune infiltration analysis of hub LMRGs

**Figure 9:**
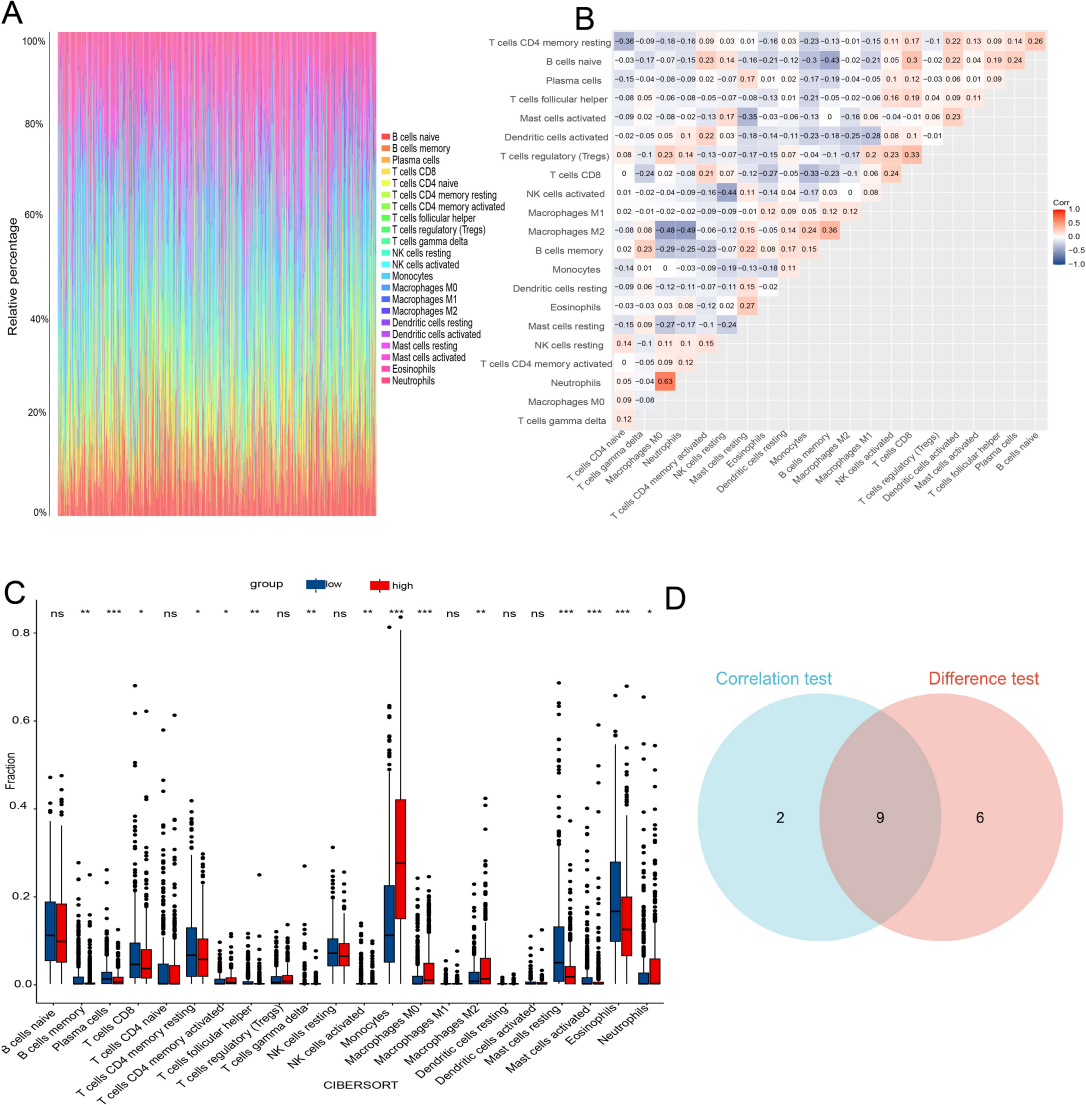
TIC profiles and correlation analysis in AML samples. **(A)** Bar graph showing the proportions of 21 TICs in AML samples. **(B)** Heatmap displaying the correlations among the 21 TICs. **(C)** Box plot comparing the proportions of the 21 immune cell types between AML samples with low and high CES1 expression levels. **(D)** Venn diagram showing the intersection of results from correlation analysis and differential analysis. * P < 0.05; ** P < 0.01; *** P < 0.001; ns, not significant.

**Figure 10:**
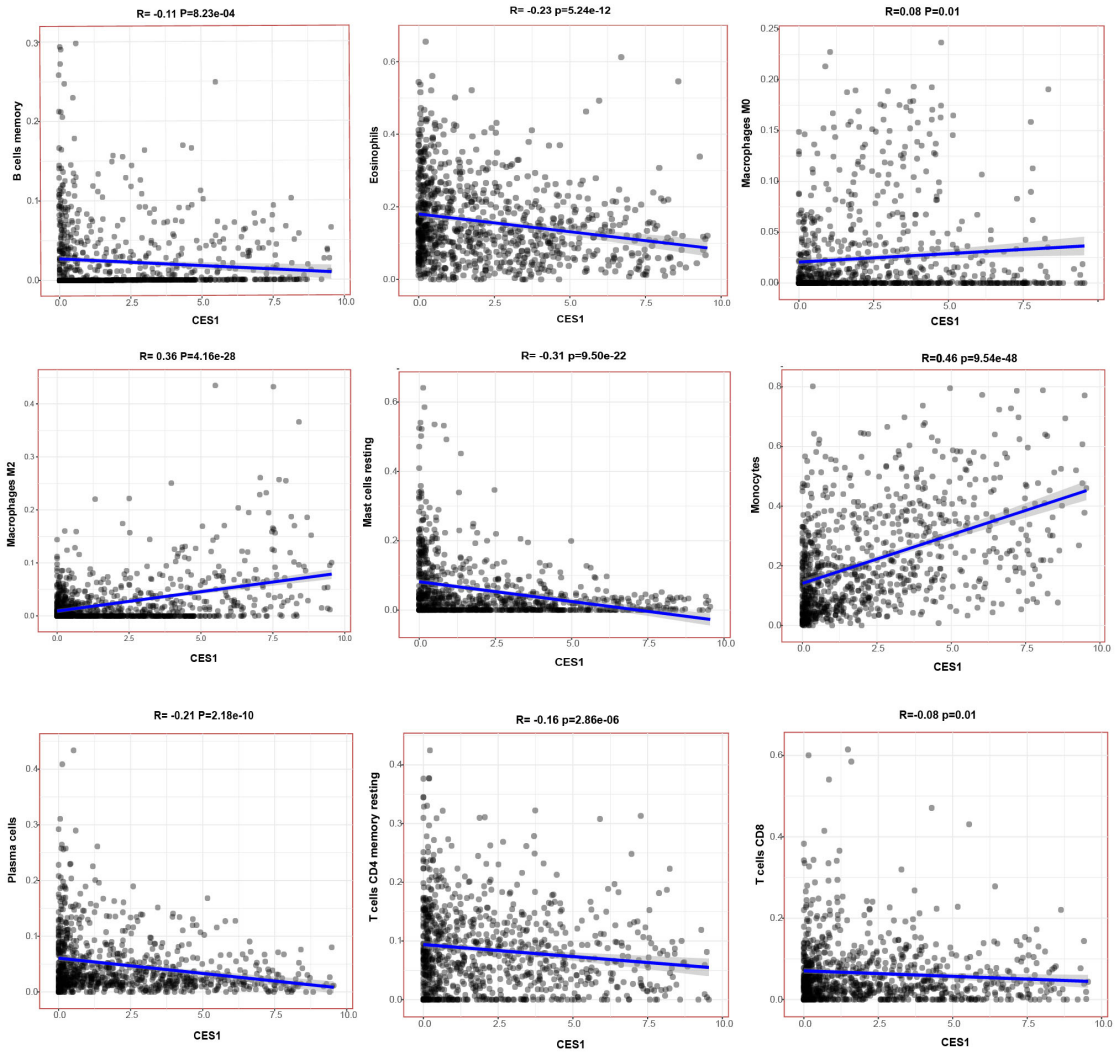
Scatterplots showing the correlation of nine TIC types significantly associated with CES1 expression (*p* < 0.05).

The CNS relapse group exhibited higher immune scores compared to the non-CNS relapse group (**Figure 8C**), suggesting changes in the immune status of the TME during CNS relapse. To further elucidate the molecular functions of CES1 in the immune system, we performed GSEA using the C7 ImmuneSigDB gene set. The results revealed significant enrichment of 3,215 gene sets in the high CES1 expression group, whereas only 15 gene sets were enriched in the low CES1 expression group. Only the top five leading gene sets are shown in **Figures 8D, E**, with each line representing a specific gene set, distinguished by different colors. The NF-kappa B and MAPK signaling pathways were also activated in the high CES1 expression group compared to the low CES1 expression group (**Supplementary Figure S4**). To further analyze the effect of CES1 on the immune microenvironment, the degree of infiltration of various immune cell types was calculated using CIBERSORT. **Figures 9A, B** display the proportions of 21 immune cell subtypes in each sample. Fifteen tumor-infiltrating immune cells (TICs) showed significant differences between the high and low CES1 expression groups (**Figure 9C**). Correlation analysis identified 11 TICs associated with CES1 expression, and further intersection revealed nine TICs affected by CES1 expression (**Figure 9D**). Specifically, we observed positive correlations between CES1 expression and monocytes, M0 macrophages, and M2 macrophages, while negative correlations were found with memory B cells, plasma cells, resting CD4 memory T cells, CD8 T cells, resting mast cells, and eosinophils (**Figure 10**). These findings suggest that CES1 may promote CNS relapse by modulating immune infiltration in the TME.

### 3.8 CES1 promotes the proliferation of AML cells and M2 macrophages

**Figure 11:**
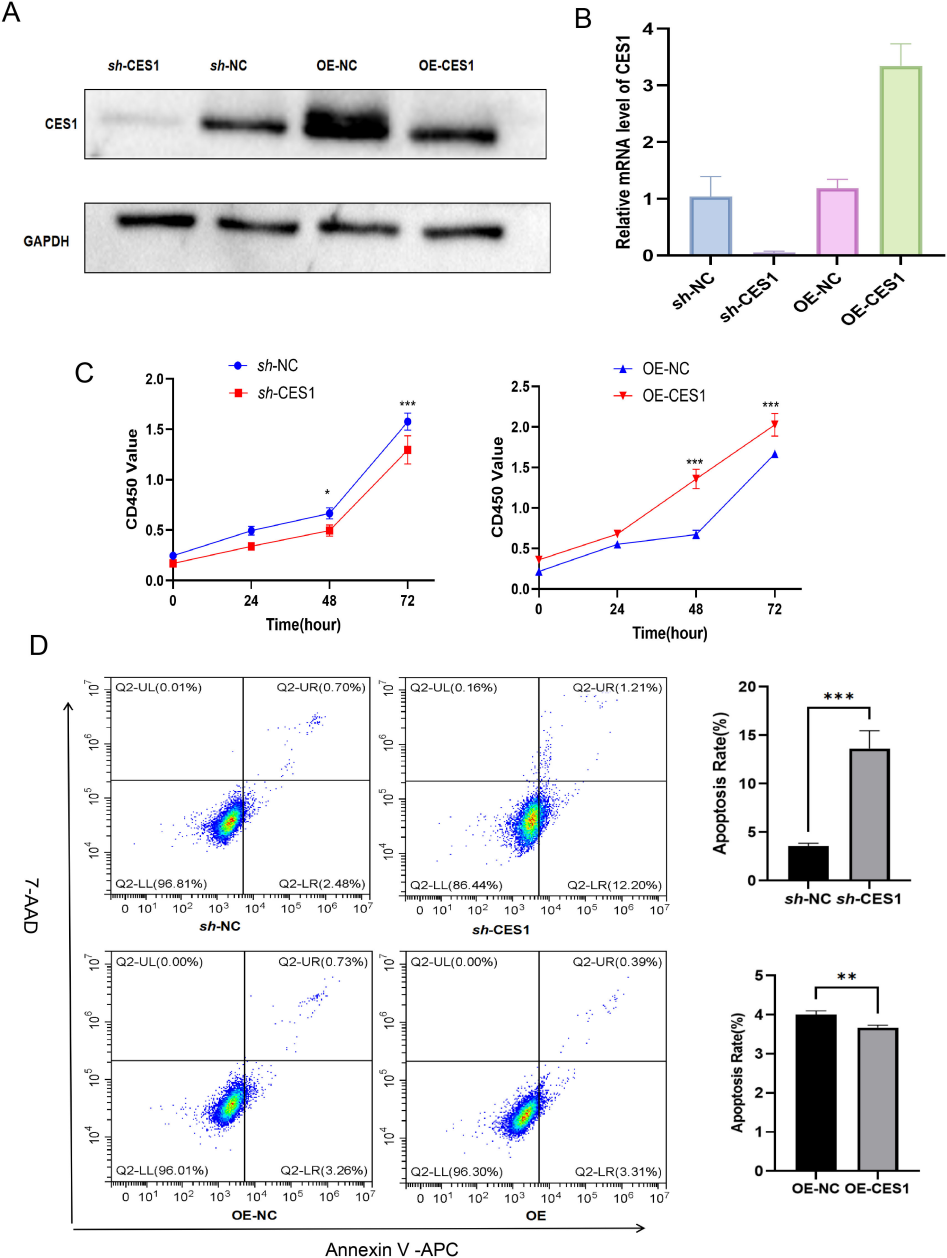
**(A)** Validation of CES1 knockdown and overexpression by Western blot. **(B)** Validation of CES1 knockdown and overexpression by RT-PCR (*n* = 3). **(C)** CCK-8 assay assessing cell viability of *sh*-NC, *sh*-CES1, OE-NC, and OE-CES1 at 0, 24, 48, and 72 h (*n* = 3). **(D)** Apoptosis analysis of *sh*-NC, *sh*-CES1, OE-NC, and OE-CES1 groups, with corresponding histograms shown on the right (*n* = 3). ^**^ *p* < 0.01; ^***^ *p* < 0.001.

**Figure 12:**
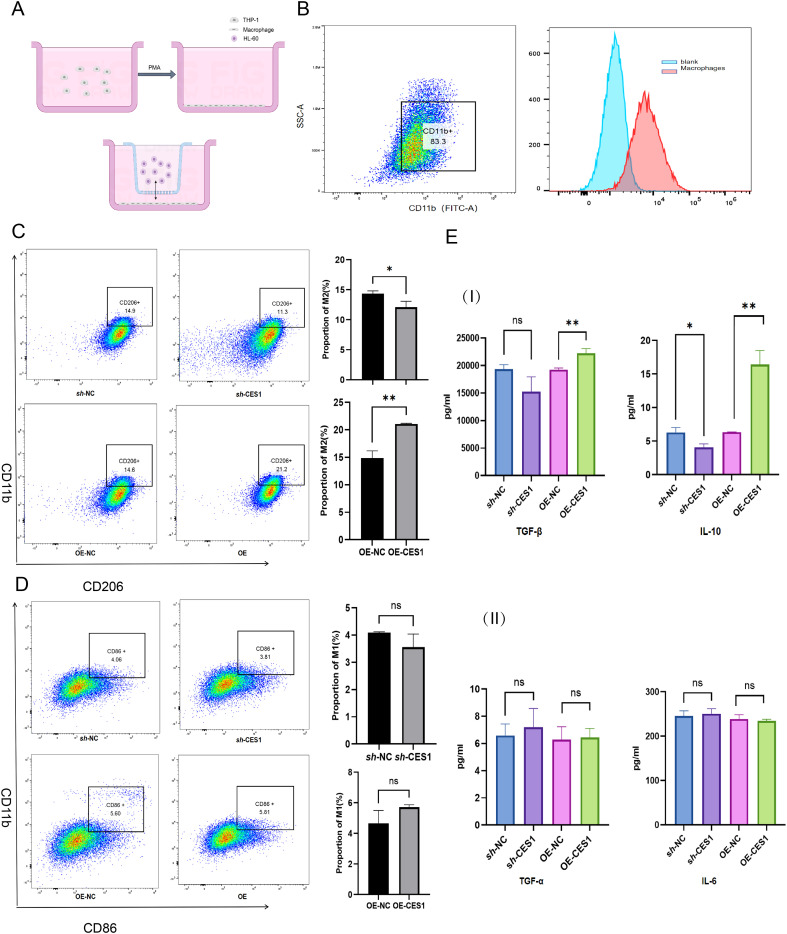
**(A)** Schematic diagram of the coculture system. **(B)** Flow cytometry results showing CD11b expression in THP-1 cells after activation into macrophages (scatterplot on left, histogram on right). **(C)** Percentage of M2 macrophages in *sh*-NC, *sh*-CES1, OE-NC, and OE-CES1 groups with corresponding statistical histograms (*n* = 3). **(D)** Percentage of M1 macrophages in *sh*-NC, *sh*-CES1, OE-NC, and OE-CES1 with corresponding statistical histograms (*n* = 3). **(E)** I: ELISA results for TNF-β and IL-10 expression in *sh*-NC, *sh*-CES1, OE-NC, and OE-CES1 groups (*n* = 3). II: ELISA results for TNF-α and IL-6 expression in *sh*-NC, *sh*-CES1, OE-NC, and OE-CES1 groups (*n* = 3). ^*^ *p* < 0.05; ^**^ *p* < 0.01; ns, not significant.

We had knocked down and overexpressed the *CES1* gene in the AML cell line (HL-60) and validated the modifications using WB and PCR (**Figures 11A, B**). The results of the CCK-8 assay and apoptosis flow cytometry showed that the OE-CES1 group exhibited increased cell proliferation (**Figure 11C**), while apoptosis was enhanced in the *sh*-CES1 group (**Figure 11D**). Given the critical regulatory role of CES1 in the TME, we investigated its influence on macrophage polarization. PMA-induced differentiation of THP1 into macrophages (with CD11b as the surface marker), which were then cocultured with HL-60 cells (**Figures 12A, B**). CD86 was used as the cell surface marker, and TNF-α and IL-6 as cytokine markers for M1 macrophages, while CD206, TNF-β, and IL-10 served as markers for M2 macrophages. Flow cytometry analysis revealed that, compared with the control group, the *sh*-CES1 group exhibited a significant decrease in CD206 expression, whereas the OE-CES1 group showed a significant increase (**Figure 12C**). No significant differences in CD86 expression were observed among the groups (**Figure 12D**). These findings were further validated by ELISA (**Figure 12E**), suggesting that CES1 can promote the proliferation of AML cells and M2 macrophage polarization. We also found that FAO levels were significantly elevated in the OE-CES1 group (**Supplementary Figure S4**), which serves as a key energy source for AML cell survival (25).

### 3.9 CES1 promotes progression of AML and M2 macrophages in xenograft tumor models

**Figure 13:**
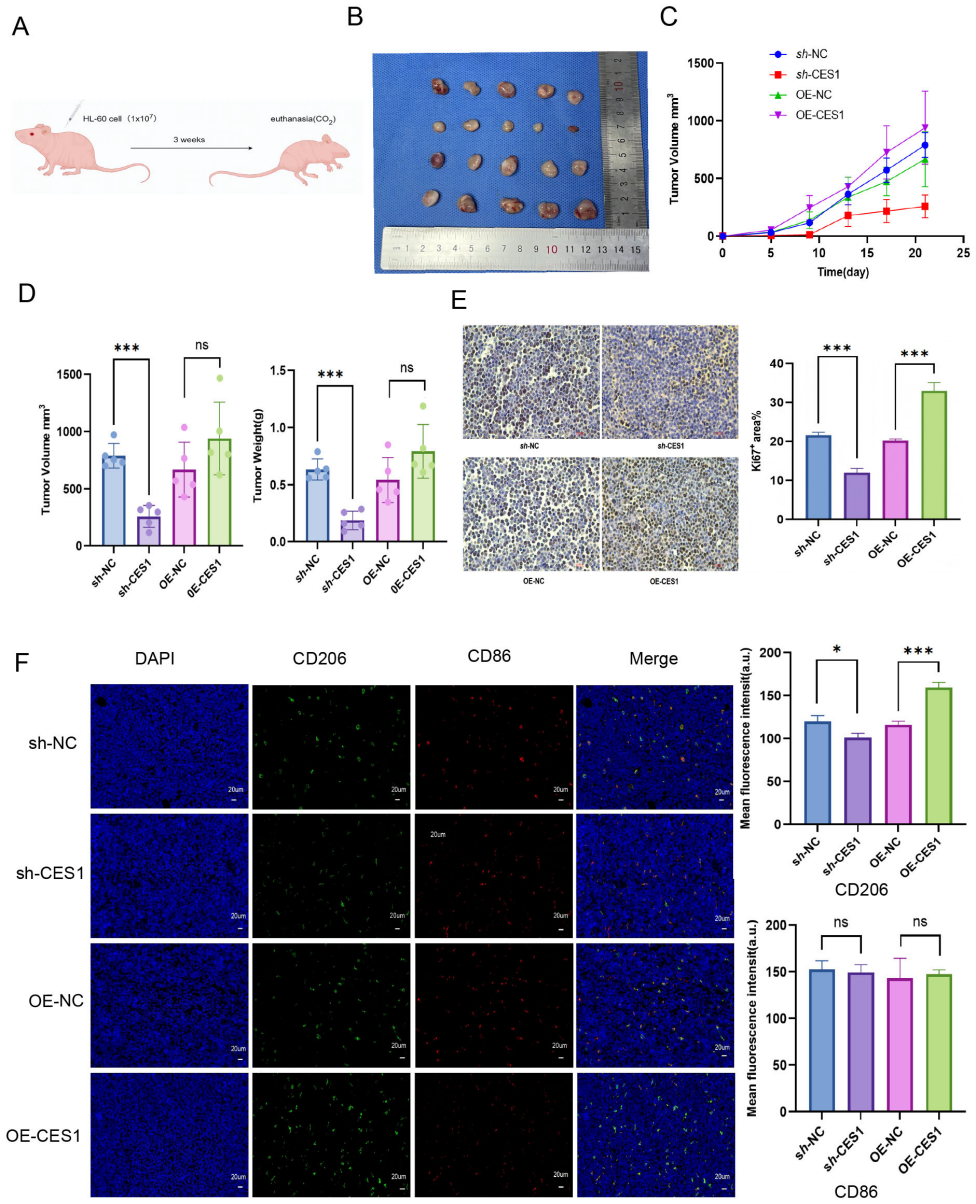
**(A)** Schematic diagram of AML xenograft tumor model construction. **(B)** Photograph of xenograft tumors from *sh*-NC, *sh*-CES1, OE-NC, and OE-CES1 groups (*n* = 5). **(C)** Tumor volumes measured at the indicated time points. **(D)** Tumor volumes and tumor weights from xenograft tumors in *sh*-NC, *sh*-CES1, OE-NC, and OE-CES1 groups. **(E)** Immunohistochemical analysis of Ki67 expression in xenografts from *sh*-NC, *sh*-CES1, OE-NC, and OE-CES1 groups (*n* = 3), with statistical histograms shown on the right. **(F)** Immunofluorescence analysis in xenografts from the s*h*-NC, *sh*-CES1, OE-NC, and OE-CES1 groups (*n* = 3), with statistical histograms shown on the right. ^*^ *p* < 0.05; ^**^ *p* < 0.01; ^***^ *p* < 0.0001; ns, not significant.

To further validate the effect of CES1 *in vitro*, we subcutaneously injected HL-60 cells treated with *sh*-NC, *sh*-CES1, OE-NC, and OE-CES1 into mice to evaluate the role of CES1 in AML xenograft tumor growth (**Figure 13A**). Tumor measurements showed that *sh*-CES1 significantly reduced both tumor volume and weight compared to *sh*-NC, whereas OE-CES1 promoted tumor growth relative to the OE-NC group (**Figures 13B–D**). Immunohistochemical analysis of Ki67 in tumor tissues showed significantly lower expression in the *sh*-CES1 group, while the OE-CES1 group exhibited higher expression (**Figure 13E**). Immunofluorescence staining further revealed contrasting CD206 levels: downregulated in the *sh*-CES1 group and upregulated in the OE-CES1 group relative to their respective controls, consistent with the *in vitro* findings. In contrast, no significant differences in CD86 expression were observed among the groups (**Figure 13F**).

## 4 Discussion

This study demonstrated that AML patients with CNS relapse exhibited higher serum lipid levels compared to those without CNS relapse. Hypercholesterolemia was identified as a risk factor for CNS relapse. Bioinformatics analyses revealed that the DEGs were involved in the regulation of lipid transport, lipid localization, and very-low-density lipoprotein particle remodeling. CES1 was identified as a hub LMRG associated with CNS relapse. GSEA enrichment and CIBERSORT analyses further indicated its role in immune cell infiltration within the immune microenvironment, especially in M2 macrophages. This was supported by functional results of CES1 knockdown and overexpression in AML cells and the AML xenograft model.

Dysregulation of lipid metabolism has been extensively studied in multiple types of cancer (26). The interplay among proteins, enzymes, and genes involved in lipid synthesis and transport can facilitate carcinogenesis and tumor progression (27). Elevated TC levels have been shown to stimulate the proliferation of hematopoietic stem cells (HSPCs) (28), which can internalize both natural and oxidized LDL-C, thereby promoting self-replication *in vitro*. In our study, we observed that LDL-C and TC levels were significantly elevated in AML patients with CNS relapse compared to those without. Hypertriglyceridemia was identified as an independent risk factor for AML CNS relapse. Abnormal lipid metabolism is also a major contributor to oxidative stress, which accelerates AML progression by increasing DNA damage and repair errors (29). Furthermore, elevated levels of TC and TG can induce an inflammatory response. This involves proinflammatory cytokines activating the MAPK signaling pathway via NF-kB-mediated metalloproteinase signaling, which in turn stimulates the release of G-CSF, IL-6, CXCL1, and other inflammatory mediators. These mediators can drive the expansion and aggregation of myeloid cells, fostering a proangiogenic and dysfunctional TME that promotes tumor growth and metastasis (30, 31). Our results also showed a negative correlation between HDL-C and WBC counts. However, the correlation was weak (*R* ^2^ = − 0.19). Although statistically significant, this suggests only a potential association between HDL-C and WBC counts. Age ≤ 45 years and WBC count ≥ 50 × 10^9^/L were influential factors for CNS relapse, consistent with previously reported findings (7). However, this study did not confirm the previously reported association between FAB subtypes M4 or M5 and CNS relapse. This discrepancy may be attributed to the limited number of patients with M4 and M5 subtypes in our cohort, highlighting the need for larger-scale studies and additional evidence.

Among the 1,368 DEGs identified between CNS relapse and non-CNS relapse, some were associated with lipid metabolism-related biological processes, including lipid transport, modulation of lipid localization, and remodeling of very-low-density lipoprotein particles. Additionally, the regulation of lipolysis in adipocytes was altered during CNS relapse. These results further support the link between lipid metabolism and AML CNS relapse.

WGCNA is a method used to analyze patterns of gene associations across samples, allowing the identification of gene modules with highly correlated expression. To identify hub genes related to lipid metabolism in CNS relapse, we intersected DEGs, LMRGs, and WGCNA-derived genes. As a result, CES1 was identified as the hub LMRG.

CES1, a member of the human carboxylesterase family, plays a role in regulating body weight and mediating lipid metabolism (32–34). CES1 has also been associated with the prognosis of various cancers, i.e., prostate cancer (35), hepatocellular carcinoma (36), and colorectal cancer (37). In our study, high CES1 expression independently predicted CNS relapse in AML. Moreover, patients with high CES1 expression had shorter overall survival compared to those with low expression. Lipid metabolic reprogramming has been reported to influence immune infiltration within the TME (38). Patients with CNS relapse had higher immune scores compared to those without CNS relapse, suggesting altered immune infiltration in the TME of CNS relapse cases. To further investigate whether CES1 also affects the TME in AML, we performed GSEA enrichment analysis using the C7 ImmuneSigDB gene set to examine the potential involvement of CES1 in immune-related pathways. Multiple immune function-related gene sets were highly enriched in the high CES1 expression group, while relatively fewer gene sets were enriched in the low CES1 expression group. These findings reflect CES1’s impact on immune function in AML. Furthermore, CIBERSORT analysis identified nine types of TICs that were correlated with CES1 expression. Thus, monitoring CES1 expression may provide insights into the immune infiltration status of the TME. Notably, a positive correlation was observed between CES1 expression and M2 macrophages. Macrophages can polarize into two distinct states depending on the stimuli: M1 (proinflammatory) or M2 (anti-inflammatory) (39). M2 macrophages can promote tumor cell migration, invasion, and distant metastasis (40, 41). AML patients with poor prognosis have been shown to exhibit an increased frequency of M2 macrophages (42). We speculated that CES1 contributes to a higher proportion of M2 macrophages, thereby promoting AML invasion. To test this, we conducted a series of experiments and observed that CES1 promotes AML progression and macrophage M2 polarization in both AML cells and AML xenograft tumor models. This validated the results of the bioinformatics analysis.

AML cells exhibit an atypical metabolic phenotype characterized by significant metabolic reprogramming, including alterations in glucose and lipid metabolism (43). AML cells promote glycolysis by activating the PI3K/AKT pathway to meet their specific energy and functional needs (44). Yang et al. (45) also proposed a novel risk model based on carbohydrate metabolism-related genes, demonstrating promising potential for prognostic classification in AML. For aberrant lipid metabolism, AML cells rely heavily on FAO for survival (25). FAO is also significantly activated in leukemia stem cells (LSCs) isolated from patients with relapsed AML (46). Notably, our study demonstrates a significant elevation of FAO in the OE-CES1 group, suggesting a potential link between CES1 and lipid metabolic regulation.

This study reveals a link between hypercholesterolemia and CNS relapse in AML, suggesting the need to monitor lipid levels and consider appropriate interventions. CES1can promote AML progression, M2 macrophage polarization, and FAO. These findings highlight CES1 as a potential therapeutic target for CNS relapse, underscoring its role in metabolic regulation and modulation of the tumor microenvironment, as well as the need for the development of novel CES1 inhibitors with improved bioavailability.

It should be acknowledged that the results of our study should be interpreted with caution due to several limitations. (1) A potential selection bias may exist due to the single-center, retrospective design. As a result, direct causal inference between lipid levels and the risk of CNS relapse remains limited. This limitation could be addressed in future studies through more rigorous inclusion criteria and multiethnic/prospective studies. (2) The sample size of AML patients with CNS relapse was small, both in the clinical cohort and in the data obtained from the TARGET database. We emphasize the need for future studies to incorporate multicenter cohorts, increase sample sizes, and validate and extend our findings. (3) There may still be some unmeasured factors (e.g., diet, lifestyle, genetic predispositions) that could influence the association between blood lipid levels and the risk of CNS relapse. (4) The exact mechanism by which CES1 modulates AML progression and M2 macrophage polarization remains unclear and warrants more in-depth investigation in future research.

## Data Availability

Publicly available datasets were analyzed in this study. This data can be found here (access link: https://ocg.cancer.gov/programs/target) and are archived in the dbGaP under accession number phs000218.
