# Supplementary material for: Hypercholesterolemia and the role of lipid metabolism gene CES1 in immune infiltration promote central nervous system relapse in acute myeloid leukemia
Source: Front Immunol. 2025 Jul 23;16:1575472. doi: 10.3389/fimmu.2025.1575472 (PMC12325197; doi:10.3389/fimmu.2025.1575472)
Supplement: Supplementary file 1 [file DataSheet1.pdf]

Supplementary Data

Supplementary Methods.
Supplementary Figures.
Figure S1. Lipid profile comparison between non-APL and APL groups.
Figure S2. lipid distributions in non-CNS relapse and CNS relapse groups in non-APL.
Figure S3. lipid distributions in non-CNS relapse and CNS relapse groups in APL.
Supplementary Tables.
Figure S4. Association of FAO , NF-κB and MAPK pathways with CES1.
Table S1. MANOVA results: Interaction Effect of APL Subtype×CNS Relapse Status on Lipid Profiles.
Table S2. The detailed lipid metabolism-related gene sets from the GSEA database.

Supplementary Methods

Cell lines and cell culture

Human AML cell line HL-60 was cultured in a medium containing 20% FBS(KEL, Shanghai) and IMDM (Solarbio, Beijing) as well as THP-1 was cultured in RPMI-1640 medium (Solarbio, Beijing) containing 15% FBS and 1% penicillin/streptomycin at 37 °C with 5% CO2.

For co culture, THP-1 was added with 100 ng/mL phorbol myristate acetate (PMA) (MCE, USA) and induced into activated macrophages after 24 hours. Subsequently, co-culture experiments were conducted with HL-60 using a transwell chamber assayll culture.

Cell transfection

The lentiviral vectors for CES1 knockdown and overexpression were purchased from GeneChem Co. Ltd. (Shanghai, China). Inoculate HL-60 (2×10^5) cells into a 6-well plate and add infection enhancing solution to the cells. Calculate the virus volume using the following formula based on the number of cell infections (MOI) and the virus titer: Virus volume (μL)= (number of cells×MOI)/virus titer. HL-60 cells were divided into the following experimental groups: Sh-CES1 group (transfected with CES1 shRNA lentiviral vector, MOI=10), Sh-NC group (transfected with control shRNA lentiviral vector, MOI=10), OE-CES1 group (transfected with CES1 overexpression lentiviral vector, MOI=30), OE-NC group (transfected with control lentiviral vector, MOI=30). The cells were incubated for 18 hours, and after 72 hours of infection, they were cultured in a medium containing 4.0 μ g/mL puromycin to screen for stable cell lines after infection.

Cell Counting(CCK8）

Using the CCK-8 kit (MCE, USA) to evaluate cell proliferation. Inoculated HL-60 cells (3×10^3 cells/well) into a 96 well plate, after incubate with CCK-8 solution at

37 ° C for 2 hours, measure the absorbance (450 nm) at 0, 24, 48, and 72 hours using an microplate reader.

Enzyme-Linked Immunosorbent Assay（Elisa）

TGFα,TGFβ， IL--6， IL-10 and fatty acid oxidation(FAO) kit was procured from Boster(China), following which it was utilised in accordance with the instructions stipulated by the reagent manufacturer.

Western blot analysis

Obtained cells protein using lysis buffer and PMSF (100mM). The protein concentration was determined by BCA. Sample protein (30 μ g) added with the same amount of loading buffer was loaded onto 10% SDS-PAGE gell and electroblotted onto 0.2 μ m PVDF membrane. After incubating PVDF membrane with 5% skim milk at room temperature for 2 hours, it was then incubated overnight with antibodies of CES1 (Zenbio, China) and GAPDH (Zenbio, China) antibodies at 4 ° C， Chemiluminescence was performed the next day after coupling with the secondary antibody.

Real-time PCR assay (RT-PCR)

Total RNA was extracted using Trizol (TRAN, China) and then reverse transcribed to cDNA by PrimeScript RT kit Perfect Real Time (Takara Bio, Japan). quantitative PCR was performed on a LightCycler 480II real-time PCR system (Roche, Switzerland). Primers were purchased from BioSune (Shanghai, China). CES1, forward primer: CAAAGACTGGGGTCTTTTGC, reverse primer: AGCCATGGTAAGATGCCTTC. GAPDH, forward primer: AGGTCGGTGTGAACGGATTTG, reverse primer: GGGGTCGTTGATGGCAACA.

Establishment of Xenograft tumours model

The animal experiments in this study have been approved by the Ethical Approval for Research Involving Animal of Nanchang Royo Biotech Co. Four-week-old BALB/c nude male mice were acclimatised to an SPF environment for a week. The mice were inoculated subcutaneously with 100 µL (1 x 10^7) liquid of sh-NC, sh-CES1, OE-NC and OE-CES1 cells. The tumour volume was measured at two-day intervals. After three weeks, the mice were euthanised. The volume of the tumour is calculated by the following formula:tumour volume (mm^3) = 0.5 × (tumour width)^2 × (tumour length)^2.The tumour weight is measured at the conclusion of the experiment.

Flow cytometry

The assessment of cell apoptosis was conducted by means of flow cytometry (Beckman) and Annexin V-APC/7AAD cell apoptosis detection kit (Elabscience China). These kits were utilised in accordance with the manufacturer's protocol to

analyse cells.Dvide the1 x 106 cells into flow tubes and treated with FcX. The samples were then subjected to incubation with 5 µl antibody of CD11b (BioLegend China), CD206-APC (BioLegend China), and CD86 (BioLegend China) at room temperature in the dark for 30 minutes for measure the expression of macrophage surface markers CD11b, CD86, and CD206. The sample measured using a flow cytometer (Beckman).

Immunohistochemical staining(IHC) &Immunofluorescence(IF)

Mouse tumor tissue was fixed with formaldehyde, embedded in paraffin, and sliced. The dewaxed sections were treated with methanol containing 3% hydrogen peroxide for 15 minutes. Wash the slices with PBS and block them with blocking serum at room temperature for 30 minutes. Then incubate the slices overnight with Ki67 primary antibody (Proteintech, USA) at 4 ° C. The next day, the primary antibody was washed away, and the slices were incubated with HRP conjugated secondary antibody at room temperature for 2 hours. Stain the cell nucleus with hematoxylin and add DAB substrate (ZSGB Bio, China) to detect proteins.

For IF staining, tissue slides were dewaxed, hydrated, and subjected to antigen repair in a sodium citrate antigen repair solution (Solarbio, China). After blocking with 10% BSA, incubate with anti-CD68, anti-CD206 antibodies, and secondary antibodies. Cell nuclei were stained with DAPI and images were obtained using confocal microscopy.

Supplementary Figures

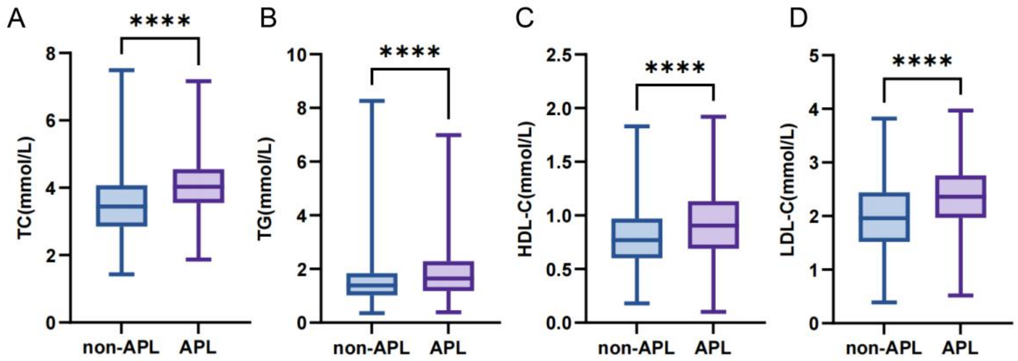

Figure S1. Lipid profile comparison between non-APL and APL groups. (A)TC levels; (B) TG levels; (C) LDL-C levels; (D) HDL-C level; **** $P < 0.0001$.

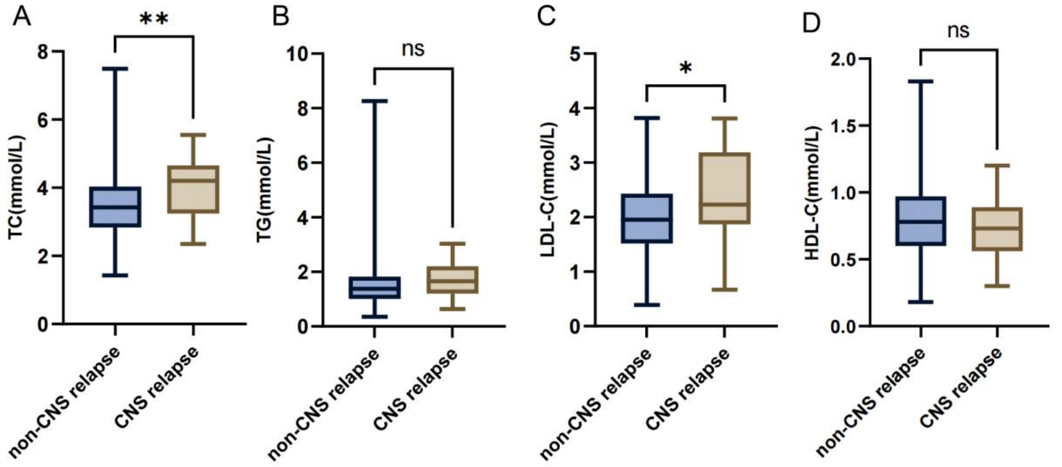

Figure S2. lipid distributions in non-CNS relapse and CNS relapse groups in non-APL. (A)TC levels; (B) TG levels; (C) LDL-C levels; (D) HDL-C level; * $P < 0.05$; ** $P < 0.01$; ns: not significant.

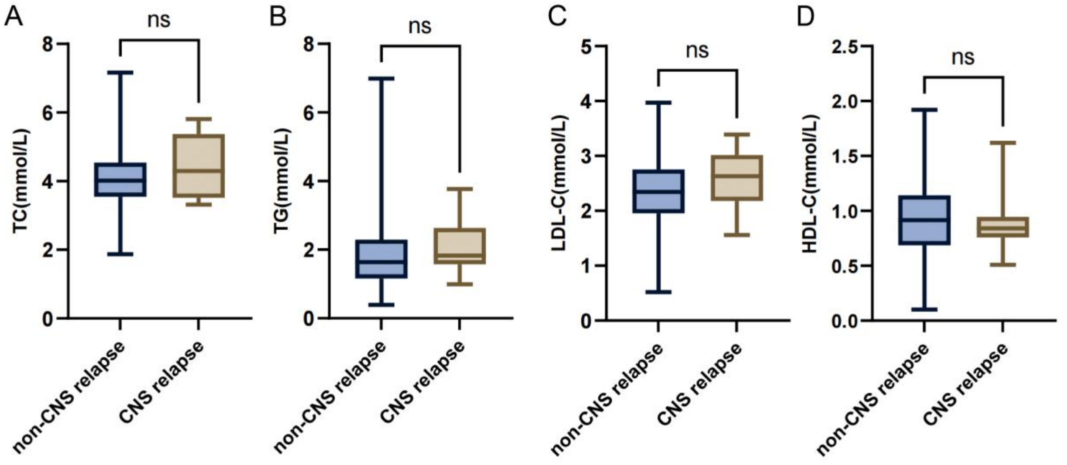

Figure S3. lipid distributions in non-CNS relapse and CNS relapse groups in APL. (A)TC levels; (B) TG levels; (C) LDL-C levels; (D) HDL-C level; ns: not significant.

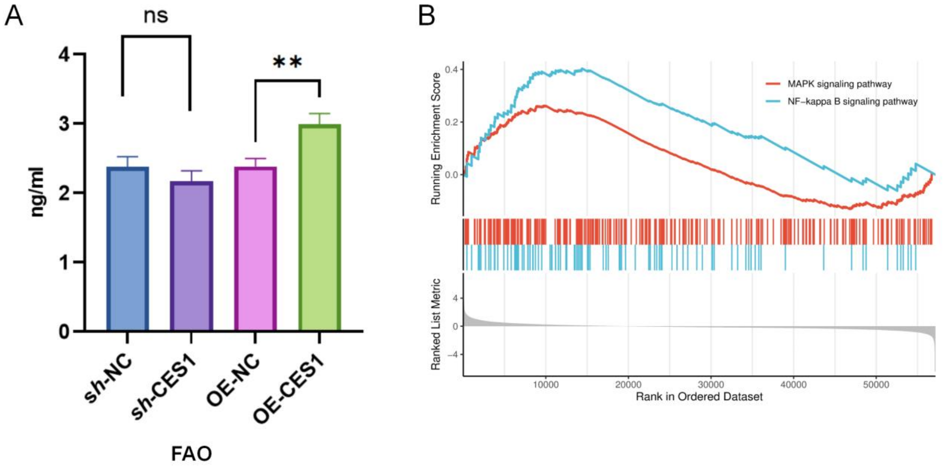

Figure S4.Association of FAO , NF-κB and MAPK pathways with CES1 (A) The expression FAO by Elisa in *sh*-NC, *sh*-CES1,OE-NC and OE-CES1, n=3. (D) Demonstrated the results of GSEA enrichment analysis, NF-κB and MAPK pathways were enriched in high CES1 expression sample . ** P < 0.01; ns: not significant.

Supplementary Tables

Table S1. MANOVA results: Interaction Effect of APL Subtype×CNS Relapse Status on Lipid Profiles.

Dependent Variables	F - statistics	P-value
TC (mmol/L)	0.388	0.534
TG (mmol/L)	0.005	0.946
HDL-C (mmol/L)	0.306	0.581
LDL-C (mmol/L)	0.354	0.552

Table S2. The detailed lipid metabolism-related gene sets from the GSEA database.

**Database**	**gene-set**
**GSEA**	REACTOME_TRIGLYCERIDE_METABOLISM
	HP_HYPERLIPOPROTEINEMIA
	HP_ABNORMAL_HDL_CHOLESTEROL_CONCENTRATION
	HP_ABNORMAL_CIRCULATING_CHOLESTEROL_CONCENTRATION
	GOERING_BLOOD_HDL_CHOLESTEROL_QTL_CIS
	HP_HYPERCHOLESTEROLEMIA
	HP_INCREASED_LDL_CHOLESTEROL_CONCENTRATION
	GOBP_REGULATION_OF_CHOLESTEROL_METABOLIC_PROCESS
	REACTOME_REGULATION_OF_LIPID_METABOLISM_BY_PPARALPHA
	REACTOME_PHOSPHOLIPID_METABOLISM
	REACTOME_KETONE_BODY_METABOLISM
	HALLMARK_FATTY_ACID_METABOLISM
	WP_LIPID_METABOLISM_PATHWAY
	REACTOME_SPHINGOLIPID_METABOLISM
	REACTOME_METABOLISM_OF_LIPIDS
	REACTOME_GLYCOSPHINGOLIPID_METABOLISM
	REACTOME_FATTY_ACID_METABOLISM
	KEGG_SPHINGOLIPID_METABOLISM
	KEGG_GLYCEROPHOSPHOLIPID_METABOLISM
	KEGG_GLYCEROLIPID_METABOLISM
	KEGG_FATTY_ACID_METABOLISM
	KEGG_PPAR_SIGNALING_PATHWAY
